# Evaluation of a simple intervention to reduce exchange transfusion rates among inborn and outborn neonates in Myanmar, comparing pre- and post-intervention rates

**DOI:** 10.1186/s12887-015-0530-5

**Published:** 2015-12-17

**Authors:** G. Arnolda, A. A. Thein, D. Trevisanuto, N. Aung, H. M Nwe, A. A. Thin, N. S. S. Aye, T. Defechereux, D. Kumara, L. Moccia

**Affiliations:** Thrive Networks, Oakland, CA USA; School of Public Health & Community Medicine, Faculty of Medicine, University of New South Wales, Sydney, NSW Australia; Department of Neonatology, University of Medicine (1), Yangon, Myanmar; Amici della Neonatologia Trentina, Trento, Italy; Children and Women’s Health Department, Medical School University of Padua, Padua, Italy; Senior Consultant Neonatologist, Central Women’s Hospital, Mandalay, Myanmar; Associate Professor, Department of Paediatrics, University of Medicine (1), Yangon, Myanmar; Senior Consultant Neonatologist, Mandalay Children’s Hospital (300), Mandalay, Myanmar; Senior Consultant Neonatologist, Central Women’s Hospital, Yangon, Myanmar; Department of Surgery, Liege University Hospital, Liege, Belgium

**Keywords:** Neonatal jaundice, Phototherapy, Exchange transfusion, Neonates, Hyperbilirubinemia

## Abstract

**Background:**

In Myanmar, approximately half of all neonatal hospital admissions are for hyperbilirubinaemia, and tertiary facilities report high rates of Exchange Transfusion (ET). The aim of this study was to evaluate the effectiveness of the pilot program in reducing ET, separately of inborn and outborn neonates.

**Methods:**

The study was conducted in the Neonatal Care Units of four national tertiary hospitals: two exclusively treating inborn neonates, and two solely for outborn neonates. Prior to intervention, no high intensity phototherapy was available in these units. Intervention in late November 2011 comprised, for each hospital, provision of two high intensity LED phototherapy machines, a photo radiometer, and training of personnel. Hospital-specific data were assessed as Relative Risk (RR) ratios comparing ET rates pre- and post-intervention, and individual hospital results were pooled when appropriate.

**Results:**

In 2011, there were 118 ETs among inborn neonates and 140 ETs among outborn neonates. The ET rate was unchanged at Inborn Hospital A (RR = 1.07; 95 % CI: 0.80–1.43; *p* = 0.67), and reduced by 69 % at Inborn Hospital B (RR = 0.31; 95 % CI: 0.17–0.57; *p* < 0.0001). For outborn neonates, the pooled estimate indicated that ET rates reduced by 33 % post-intervention (RR_MH_ = 0.67; 95 % CI: 0.52–0.87; *p* = 0.002); heterogeneity was not a problem.

**Conclusion:**

Together with a photoradiometer and education, intensive phototherapy can significantly reduce the ET rate. Inborn Hospital A had four times as many admissions for jaundice as Inborn Hospital B, and did not reduce ET until it received additional high intensity machines. The results highlight the importance of providing enough intensive phototherapy units to treat all neonates requiring high intensity treatment for a full course.

**Trial registration:**

Australian New Zealand Clinical Trials Registry ACTRN12615001171505, 2 November 2015.

## Background

Neonatal jaundice is found in about 60 % of term and 80 % of preterm neonates in the first week of life [1], because neonates produce bilirubin at unusually high rates, and are inefficient at metabolising and excreting it [2]. This ‘physiological jaundice’ usually resolves spontaneously, and can be differentiated from a number of pathological conditions (e.g., Rh (D) isoimmunisation, ABO incompatibility) which result in extreme hyperbilirubinaemia [3]. Blood exchange transfusion (ET) is a common intervention to treat extreme hyperbilirubinaemia with the goal of preventing bilirubin encephalopathy and death [4], rapidly removing about 50 % of the circulating bilirubin [5]. While effective, ET is associated with a range of procedure-related risks of mortality and morbidity [4].

We are not aware of any estimates of national ET rates, but hospital-based studies provide some guide. A Greek study reported the ET rates in an Athens maternity hospital during 1957–1961 at 435 ET per 100,000 live births and, after the introduction of routine phototherapy, a much lower rate of 50 ET per 100,000 live births in a separate Athens maternity hospital in 1980–1992 [6]. Similarly, the ET rate among inborn neonates at a single hospital in the USA during the period 1986–2006 averaged an estimated 74 neonates per 100,000 live births, with a statistically significant reduction in exchange transfusion incidence over time [7]. The reduction in ET in industrialised countries can be attributed to a number of factors, including screening for ABO and Rh blood types and obstetric or neonatal treatment [7], early neonatal screening for jaundice, and the adoption of phototherapy as a means of preventing exchange transfusion [8].

In Myanmar, the National Hospital Statistics Report for 2011 reveals that admissions relating to neonatal jaundice are responsible for 46 % of all hospital admissions for conditions originating in the perinatal period [9]. While we are not aware of national data on ET rates in Myanmar, internal data from two tertiary maternity hospitals (the Central Women’s Hospitals of Yangon and Mandalay; ‘Inborn Hospitals A and B’) and two tertiary paediatric hospitals (Yangon Children’s Hospital and Mandalay 300-bedded Children’s Hospital; ‘Outborn Hospitals A and B’) showed unexpectedly high rates of ET. A pilot program was implemented at these four hospitals, to improve treatment of neonates admitted to the Neonatal Care Units (NCUs).

The aim of this study was to evaluate the effectiveness of the pilot program in reducing ET, separately of inborn and outborn neonates.

## Methods

### Setting and context

Inborn Hospitals A and B are both national tertiary obstetric referral hospitals that only admit inborn neonates, while Outborn Hospitals A and B are both tertiary paediatric referral hospitals that only admit outborn neonates. Prior to 2014, all four hospitals had NCUs which provided care at American Academy of Pediatrics [AAP] Level 2B [10].

All hospitals used conventional blue-light phototherapy machines (Philips 20 W/52 blue tubes or Yondon 20 W YD-FL-20W) at the time of intervention. Despite the fact that hospital staff attempted to replace lights regularly, the lack of a photo radiometer meant that clinicians were frequently uncertain as to the quality of treatment they were providing. The irradiance of some of the conventional phototherapy machines was measured using a photo radiometer (BLMv7; Medical Technology Transfer and Services [MTTS], Hanoi, Vietnam) in June 2011. Results are shown in Table 1 separately for single and double-sided machines; the irradiance and was in the range 5–23 μW/nm/cm^2^.

**Table 1:** Irradiance of conventional blue light phototherapy machines prior to intervention

Hospital	Single or double-sided	No. machines	No. measured	Median readings [Range] µW/nm/cm^2^
Inborn A	Single	6	6	12.0 [8–23]
	Double - above	2	2	15.0 [10–20]
	Double - below			15.0 [10–20]
Inborn B	Single	7	7	7.0 [5–11]
	Double - above	2	2	11.0 [5–17]
	Double - below			12.5 [8–17]
Outborn A	Single	9	3	20.0 [11–23]
	Double - above	1	1	10.0 [n/a]
	Double - below			10.0 [n/a]
Outborn B	Single	10	4	11.0 [7–13]
	Double - above	3	0	n/a
	Double - below			

### Intervention

In November 2011, each hospital participated in an intervention funded and implemented by the Breath of Life (BOL) program of Thrive Networks, an international non-governmental organization. The intervention comprised provision of NCU equipment, including two LED phototherapy machines (MTTS PTV3000) and a photo radiometer (MTTS BLMv7), and training from one of the authors (DT) covering a variety of clinical subjects including management of neonatal hyperbilirubinaemia and phototherapy. The MTTS PTV3000 provides phototherapy from above, using Philips ‘Luxeon Rebel Royal Blue’ LED bulbs with a peak wavelength of 455 nm, calibrated at shipping to a maximum irradiance of 40 μW/nm/cm^2^ as measured at 40 cm. Calibration was done using the MTTS BLMv7, which has a sensor element with a half power response band from 420 to 505 nm (TCS3404CS, Taos Inc., Austria) [11] and a filter with a half power response band from 441 to 466 nm [#071 ‘Tokyo Blue’, Filters Op Maat, Netherlands] [12]. The Philips LED bulbs have an expected life (70 % lumen maintenance) of 50,000 h [13].

Training encouraged use of the AAP 2004 Guidelines for management of hyperbilirubinaemia in neonates born at 35 weeks gestation and above [14]; for neonates born <35 weeks gestation, training promoted use of the phototherapy treatment and exchange transfusion thresholds recommended by the UK National Institute for Clinical Excellence [1]. These were the guidelines already in use at the Central Women’s Hospital, Yangon, which is the hospital responsible for setting policy on facility based neonatal care in Myanmar. Hospital staff attended a 4 h meeting to provide training in management of hyperbilirubinaemia and use of the equipment, and to establish a register to record data on neonates treated on the LED phototherapy machines.

In November and December 2012, additional MTTS PTV3000 LED machines were donated to each of the hospitals; three each to Inborn Hospital A and Outborn Hospital A, and one each to Inborn Hospital B and Outborn Hospital B.

### Data sources for comparing pre- and post-intervention periods

For the purposes of evaluation we defined the pre-intervention period as calendar 2011 and the post-intervention period as calendar 2012. While not precisely aligned with the date of intervention (late November 2011), the misclassification was considered minor. This definition permitted the use of NCU Annual Reports as the main data sources for the evaluation in three of the four hospitals. The Annual Reports provided information on the number of: live births (inborn); NCU admissions for any reason; NCU admissions specifically for jaundice or, at Inborn Hospital A where admission for jaundice was not recorded, NCU admissions treated with phototherapy; and ETs.

The Annual Reports at Outborn Hospital A did not contain the relevant data, so de-identified data were retrospectively collected from the NCU Admission Register (total admissions and admissions for jaundice) and the ET Register (number of ETs); as the available 2011 ET Register only commenced in mid-June 2011 (the previous register was not locatable), the pre-intervention period for this hospital was defined as July–December 2011, and the post-intervention period as July–December 2012.

### Data source for information on neonates treated on LED in the post-intervention period

To assist with understanding the intervention, all four hospitals agreed to collect a limited amount of information on each neonate treated, during project implementation. An LED Treatment Register was established, including: age at admission (days); Total Serum Bilirubin (TSB) at admission; duration of LED phototherapy treatment in days (date of end of treatment – date at start); and TSB prior to exchange transfusion. The LED Treatment Register data was only available following intervention, and for different durations at each hospital, ranging from 12 to 20 months; at three hospitals reporting was continuous, stopping at different dates, but in the fourth (Outborn Hospital A) there were gaps in reporting.

TSB readings at the four facilities could be processed in a variety of locations: the NCU; the hospital laboratory; or an external laboratory. In three of the four hospitals, the vast majority of tests were performed on the NCU bilirubin meter, while in the fourth (Outborn Hospital B) most readings were performed in the hospital laboratory. At one of the hospitals (Outborn Hospital A), the NCU bilirubin reader had a maximum reading of 30 mg/dL (513 μmol/L); no other Bilirubin analyser had a maximum reading.

### Ethical approval

Retrospective ethics clearance was sought and received from the Ethical Committee on Medical Research Involving Human Subjects, Department of Health, Myanmar (approval #14/2014), before any publication. As the study was an evaluation comparing outcomes before and after routine implementation of an evidence-based facility-level intervention, approved retrospectively, the Ethics Committee did not require informed parental consent.

### Analysis

Relative Risk ratios (RRs) were calculated by comparing pre- and post-intervention ET rates. Inborn and Outborn hospitals were analysed separately and the two hospitals in each group were treated as separate strata. Heterogeneity was assessed by examining Cochran’s Q and the I^2^ statistic [15], with an I^2^ value of >40 % taken to indicate important levels of heterogeneity. If important heterogeneity was identified, hospital RRs were reported individually and sources of heterogeneity explored. If heterogeneity was not identified, hospital-specific RRs were pooled using the Mantel-Haenszel method, assuming a fixed-effects model. Assessments of heterogeneity and pooling of data, where appropriate, were performed in RevMan 5.3 [16].

## Results

### Pre- and post-intervention data

Table 2 shows key characteristics of the two maternity hospitals, and Table 3 shows key characteristics of the two paediatric hospitals.

**Table 2:** Characteristics of two tertiary Myanmar maternity hospitals, in 2011 and 2012

		Inborn Hospital A		Inborn Hospital B	
		2011	2012	2011	2012
Live births		7509	8720	5696	5369
NCU admissions		1102	1259	1678	1644
NCU admission rate		14.7 %	14.4 %	29.5 %	30.6 %
Admissions for Jaundice^a^		747	832	242	220
% of NCU admissions		67.8 %	66.1 %	14.4 %	13.4 %
Estimated number of phototherapy machines^b^	Conventional – single sided	6		7	
	Conventional – double sided	2		2	
	LED	From November 2011: 2		From November 2011: 2	
		From November 2012: 5		From December 2012: 3	
Date of intervention		23 November, 2011		21 November, 2011	

^a^At Inborn Hospital A this was actually infants treated with phototherapy, rather than being admissions for jaundice

^b^Number of conventional machines are as recorded as hospital visit in June 2011 – this number was not subsequently monitored, but is not believed to have changed markedly in 2012

**Table 3:** Characteristics of two tertiary Myanmar paediatric hospitals, in 2011 and 2012

		Outborn Hospital A		Outborn Hospital B	
		2011	2012	2011	2012
NCU admissions		1363	1408	765	492
Admissions for Jaundice		363	366	281	135
% of NCU admissions		26.6 %	26.0 %	36.7 %	27.4 %
Estimated number of phototherapy machines^a^	Conventional – single sided	9		10	
	Conventional – double sided	1		3	
	LED	From November 2011: 2		From November 2011: 2	
		From November 2012: 5		From December 2012: 3	
Date of intervention		23 November, 2011		21 November, 2011	

^a^Number of conventional machines are as recorded as hospital visit in June 2011 – this number was not subsequently monitored, but is not believed to have changed markedly in 2012

In 2011, there were 118 ETs among inborn neonates at the two maternity hospitals, decreasing to 94 ETs in 2012 (Table 4). At Inborn Hospital A, the ET rate among neonates admitted for jaundice was 10.0 % pre-intervention and 10.7 % post-intervention (RR = 1.07; 95 % CI: 0.80–1.43; *p* = 0.68). At hospital B, by contrast, the intervention rate reduced dramatically from 17.8 % pre-intervention to 5.5 % post-intervention, resulting in a 69 % relative reduction in ET rates (RR = 0.31; 95 % CI: 0.17–0.57; *p* < 0.0001). The presence of substantial heterogeneity (I^2^ = 92 %) prevented the pooling of these data.

**Table 4:** Relative risk of ET in two tertiary Myanmar maternity hospitals, in 2011 and 2012

Hospital	Period	Admissions for jaundice	ET	% ET	RR [95 % CI]	RR_MH_ [95 % CI]
Inborn Hospital A	2011	747	75	10.0 %	1.07 [0.80–1.43]	nc^a^
	2012	832	82	10.7 %		
Inborn Hospital B	2011	242	43	17.8 %	0.31 [0.17–0.57]	
	2012	220	12	5.5 %		

*RR* Relative Risk ratio, *RR*_*MH*_ Mantel-Haenszel pooled RR

^a^RR_MH_ not calculated due to extreme heterogeneity (I^2^ = 92 %)

The NCU Director of Inborn Hospital A revealed that during 2012 jaundiced patients were being removed from the LED machines before treatment was complete, to try and offer the benefit of the higher irradiance to as many neonates as possible; we were informed that this practice stopped after an additional three LED machines were provided in November 2012. We therefore decided to undertake a *post hoc* analysis, comparing 2013 ET data with the 2011 (baseline) data, to explore the possible impact of additional machines. The results or the *post hoc* analysis, are shown in Table 5: the 2013 ET rate at Inborn Hospital A reduced to 3.2 %, leading to a 68 % relative reduction in ET rates (RR_2013 vs 2011_ = 0.32; 95 % CI: 0.21–0.48; *p* < 0.0001), while the 2013 ET rate at Inborn Hospital B was 7.8 % resulting in a 56 % relative reduction (RR_2013 vs 2011_ = 0.44; 95 % CI: 0.25–0.77; *p* < 0.0001). As heterogeneity was no longer observed, a pooled result was calculated, showing a reduction of ET by 64 % among inborn neonates (RR_MH 2013 vs 2011_ = 0.36; 95 % CI: 0.26–0.49; *p* < 0.0001). Relevant to this analysis, we note that Inborn Hospital A also received 7 Lullaby LED Phototherapy Units [GE Healthcare, Maryland, USA] [17] in November 2013, overlapping the end of the second post-intervention period.

**Table 5:** Relative risk of ET in two tertiary Myanmar maternity hospitals, with 2013 as post-intervention period

Hospital	Period	Admissions for jaundice	ET	% ET	RR [95 % CI]	RR_MH_ [95 % CI]
Inborn Hospital A	2011	747	75	10.0 %	0.32 [0.21–0.48]	0.36 [0.26–0.49]
	2013	968	31	3.2 %		
Inborn Hospital B	2011	242	43	17.8 %	0.44 [0.25–0.77]	
	2013	192	15	7.8 %		

*RR* Relative Risk ratio, *RR*_*MH*_ Mantel-Haenszel pooled RR

Among outborn neonates, there were 140 ETs in 2011 and 47 in 2012. As shown in Table 6 the ET rate at Outborn Hospital A reduced from 31.7 % of admissions for jaundice in 2011 to 19.2 % in 2012 (RR = 0.61; 95 % CI: 0.42–0.87; *p* = 0.008). At Outborn Hospital B, the ET rate reduced from 29.2 % in 2011 to 21.5 % in 2012 (RR = 0.74; 95 % CI: 0.51–1.07; *p* = 0.10). As there was limited heterogeneity the pooled result was calculated, leading to an overall estimate of a 33 % relative reduction in ET rates across the two paediatric hospitals (RR_MH_ = 0.67; 95 % CI: 0.52–0.87; *p* = 0.002).

**Table 6:** Relative risk of ET in two tertiary Myanmar paediatric hospitals, in 2011 and 2012

Hospital	Period	Admissions for jaundice	ET	% ET	RR [95 % CI]	RR_MH_ [95 % CI]
Outborn Hospital A	2011^a^	183	58	31.7 %	0.61 [0.42–0.87]	0.67 [0.52–0.87]
	2012^a^	182	35	19.2 %		
Outborn Hospital B	2011	281	82	29.2 %	0.74 [0.51–1.07]	
	2012	135	12	21.5 %		

*RR* Relative Risk ratio, RR_MH_ Mantel-Haenszel pooled RR

^a^Outborn Hospital A data restricted to the 6 months July–December of these years, as ET Register unavailable prior to July 2011

### Post-intervention data on LED treated neonates

Selected characteristics of neonates treated on the LED phototherapy machines are shown at Table 7 Conventional blue-light phototherapy machines were also used during this period, but clinicians indicated that the LED machines were preferentially allocated to neonates with higher TSB.

**Table 7:** Characteristics of neonates treated for jaundice with LED machines post-intervention

	Inborn		Outborn	
	Hospital A	Hospital B	Hospital A	Hospital B
Months reported^a^	13	19	12	20
Neonates treated on LED	433	266	339	251
Infants treated/month^a^Median [IQR]	32 (25–49)	12 (10–17)	30 (19.5–34.5)	10.5 (8.5–15)
Age at admission (days)^b^: Median [IQR]	2.0 [2.0–4.0]	2.0 [1.0–3.0]	3.0 [2.0–5.0]	3.0 [2.0–5.0]
TSB at admission^c^ (μmol/L): Median [IQR]	301 [255–347]	249 [211–280]	325 [234–473]	311 [224–445]
Admitted with EH^c^: n (%)	12 [2.8 %]	11 [4.3 %]	105 [31.6 %]	59 [26.8 %]
Of those with EH, number given ET: n (%)	11 [91.7 %]	5 [45.5 %]	70 [66.7 %]	23 [39.0 %]
Age at admission of infants with EH (days): Median [IQR]	3.0 [2.0–4.5]	2.0 [2.0–4.0]	4.0 [2.0–5.0]	3.0 [2.0–4.0]
Age at admission of infants without EH (days): Median [IQR]	2.0 [2.0–4.0]	2.0 [1.0–3.0]	3.0 [2.0–5.0]	3.0 [2.0–5.0]
Duration of phototherapy (days)^d^: Median [IQR]	1.0 [1.0–1.0]	2.0 [1.0–2.0]	1.0 [1.0–2.0]	2.0 [1.0–2.0]
TSB at ET^e^ (μmol/L): Median [IQR]	393 [335–431]	405 [362–456]	500 [475–513]	474 [416–539]

*ET* Exchange Transfusion, *EH* Extreme Hyperbilirubinaemia (TSB >428 μmol/L), *IQR* Interquartile range

^a^Outborn Hospital A reported 12 months of data, spread over 20 months from late November 2011 to June 2013, due to staffing interruptions. The other three hospitals reported data for consecutive month. Data at Inborn Hospital A were partial for the first and last of 14 reporting months, leading to an estimated 13 reporting months; the median and IQR exclude the first and last month

^b^Age at admission was missing for one record at Inborn Hospital A and four records at Inborn Hospital B

^c^TSB at admissions was missing for eight records at Inborn Hospital B, seven at Outborn Hospital A, and 31 at Outborn Hospital B

^d^Duration of phototherapy was missing for 1 neonate at Inborn Hospital A, 2 at Inborn Hospital B, 84 at Outborn Hospital A and 22 at Outborn Hospital B

^e^TSB at ET was missing for 5 of 64 transfused neonates at Inborn Hospital A, 5 of 18 transfused neonates at Inborn Hospital B, 42 of 88 transfused neonates at Outborn Hospital A, and 12 of 38 transfused neonates at Outborn Hospital B; note that most readings at Outborn Hospital A were performed on equipment with a maximum possible reading of 513 μmol/L

Median age at admission was 2 days for neonates admitted to the inborn NCUs compared to 3 days in the two outborn NCUs. While the median TSB at admission was slightly lower for the inborn neonates (301 and 249 μmol/L) than the outborn neonates (325 and 311 μmol/L), there is a marked difference in the distributions. The proportion of neonates admitted with extreme hyperbilirubinemia (TSB > 427 μmol/L) was 3.7–4.3 % of inborn neonates, in comparison to 28–32 % among outborn neonates.

Of those with extreme hyperbilirubinaemia, the proportion who had an ET varied enormously: 92 % and 45 % in the two maternity hospitals, and 67 % and 39 % in the two paediatric hospitals. The median age at admission of neonates with extreme hyperbilirubinaemia was 2–3 days for the two maternity hospitals, and 3–4 days for the two paediatric hospitals, and was not markedly later than for neonates without extreme hyperbilirubinaemia at admission (median of 2 days at both maternity hospitals, and 3 days at both paediatric hospitals). The only statistically significant difference was in Outborn Hospital A, where neonates with extreme hyperbilirubinaemia were admitted at a median of 4 days compared to 3 days for neonates without extreme hyperbilirubinaemia (*p* = 0.03 by Kruskal-Wallis test).

The duration of LED phototherapy was a median of 1 day at Inborn Hospital A and Outborn Hospital A, and 2 days at the Inborn Hospital B and Outborn Hospital B. TSB at ET was available for a subset of neonates that received LED phototherapy and went on to receive a transfusion. Median TSB at ET was noticeably lower among the inborn neonates having ETs (393 and 405 μmol/L) than the outborn neonates (500 and 474 μmol/L).

## Discussion

While ET is valuable for preventing bilirubin encephalopathy, the procedure is itself associated with mortality and morbidity and should be avoided whenever possible. In high resource settings, estimates of procedure related mortality from the 1950s to the 1970s, before it was established that phototherapy was effective at reducing ET, ranged from 3.7/1000 to 32.0/1000 ETs [18–24]. In low resource settings, where ET is still required at least in part due to unavailability of intensive phototherapy, results have been reported ranging from 0 to 182 deaths/1000 ETs [25–30]. The commonest morbidities associated with ET are thrombocytopenia and hypocalcaemia in both high [7, 31] and low resource settings [4, 25–28].

Phototherapy has been proven by randomised trial to markedly reduce the need for ET in high resource settings [8], so the current study merely seeks to quantify the reduction possible in a low resource setting. The current study confirms that simple provision of LED phototherapy, a photo radiometer, and provision of standardised training in use of existing guidelines, can result in a 33 % reduction in ET among outborn neonates, and a reduction of ET of 68 % at one hospital treating inborn neonates, and no reduction at the other.

In exploring this heterogeneity in the inborn hospital results, clinicians stated that patients were being removed from the LED phototherapy machines early in an attempt to share the high intensity treatment among as many patients as possible, as there were too many patients to be treated. A supplementary, *post hoc*, analysis was therefore defined comparing a period after additional LED machines had been provided to the inborn hospitals (2013); there was no heterogeneity in this analysis, and it suggested a reduction of 64 % among inborn neonates across the two hospitals. Caution must be taken in interpreting this result, because the time periods was defined *post hoc*, but the absence of heterogeneity in the results across the two inborn NCUs, and its conformance with randomised trial results, suggest that it is plausible. Nevertheless, it is also plausible that other relevant factors, the specifics of which we are unaware, also changed.

### Differential impact among inborn vs outborn neonates

The estimated relative reduction in ET at one maternity hospital in the formal analysis, and both in the supplementary period, was twice that found in paediatric hospitals. Data from the LED Treatment Registers show that that the median TSB at admission is roughly similar for both inborn and outborn neonates, but the distributions are strikingly different, with almost a third of outborn neonates admitted with extreme hyperbilirubinaemia in comparison to just 4 % of inborn neonates.

Late admission of outborn neonates has been reported in many case series. For example, one Nigerian case series found that 25 of 28 neonates admitted with Acute Bilirubin Encephalopathy (ABE) were outborn [32], another found that all six neonates with kernicterus were outborn [33], and a third reported that 26 of 27 cases of ABE were outborn – the sole inborn neonate to develop ABE followed parental refusal of ET [34]. When neonates are admitted with signs of ABE, prompt ET is recommended [14].

The results in the paediatric hospitals demonstrate that the simple interventions reported here can have a positive impact, but the extent of that impact may be limited by the ‘late’ presentation of many outborn neonates. In only one of the four hospitals does the data show that neonates presenting with extreme hyperbilirubinaemia at admission were older than neonates presenting with lower levels of hyperbilirubinaemia; thus ‘late presentation’ must be defined in terms of the severity of illness at the time of hospital presentation, rather than age of the neonate in days. This can only be addressed by additional interventions to ensure that parents are educated to identify rapidly developing jaundice, and that neonates are regularly and routinely assessed and promptly referred for treatment when appropriate. It is estimated that 36 % of births in Myanmar deliver in facilities [35]; as a substantial proportion of the facility-born neonates are promptly discharged home, education of parents and community-based intervention represents a major undertaking.

Finally, it should be noted that neither maternity hospital NCU accepted inborn neonates back into the nursery after they were discharged; if inborn neonates required treatment for jaundice after discharge from the hospital, they had to be admitted to a separate hospital as an outborn admission. Thus any ETs avoided by inborn neonates in this study were ETs during the birth admission. This policy was unchanged between the two study periods and does not therefore alter our conclusions about the potential impact of the intervention on ET rates.

### Which intervention elements were important?

The current study was implemented as operational research, exploiting readily available or relatively easily collected data to evaluate routine roll-out of an evidence-based intervention. As a result, we are unable to comment formally about which element(s) of intervention are most important, but can only explore the evidence. There are three elements of intervention: provision of LED phototherapy machines; provision of a photo radiometer; and provision of training using standard guidelines. We will consider the potential contribution of each of the elements individually but, as noted below, we believe that all three elements are essential components of any intervention to reduce ET.

#### Role of LED Phototherapy machines

Prior to the provision of LED phototherapy, none of the phototherapy machines in use at the four pilot hospitals was providing ‘intensive phototherapy’ (>30 μW/nm/cm^2^) as defined in the 2004 AAP Guidelines [14]. The provision of LED machines delivering irradiance of 40 μW/nm/cm^2^ represented an increase of irradiance by a factor of 2–5 times that previously available on the conventional blue light machines used at the four hospitals. This increase in irradiance is to some extent offset by the increase in surface area exposed by the use of double-sided phototherapy machines, at least one of which was available in each of the four NCUs.

Two LED phototherapy machines were provided to each of the four hospitals. This number of machines was adequate to facilitate reduction in ET in the three hospitals with fewer admissions for jaundice (135–366 admissions for jaundice in the post-intervention period; one LED machine per 68–183 jaundiced neonates), but inadequate for a hospital with a high volume of jaundiced neonates (832 treated neonates; one LED machine per 416 treated). While we would have anticipated partial reduction in ET rates at this hospital, no reduction was observed, and we interpreted this as due to the early cessation of treatment to permit more babies to be treated with LED. When the number of LED machines was increased to 5 (968/5 = 194 treated neonates per LED machine at Hospital), the ET rates reduced sharply; this suggests that one LED machine is required per 150–200 admissions for jaundice.

#### Role of photo radiometer

None of the four hospitals had a photo radiometer prior to the intervention, and clinicians did not therefore know the irradiance being provided by each machine. In addition, very few of the conventional phototherapy machines in use prior to the intervention had counters which recorded cumulative hours of use, to facilitate timely bulb replacement, in line with manufacturers’ specifications (usually 1500 or 2000 h of use). In the absence of counters, staff can only know when to change the blue light tubes if they carefully log the number of hours of use of each machine, to ensure that bulbs are changed at the appropriate time.

In Myanmar and elsewhere, the BOL program has noted that simple provision of a photo radiometer often leads to rapid improvement in the average irradiance of the conventional machines (due to timely replacement of tubes) and to triaging of the neonates with the highest TSB to the machine with the highest irradiance. We did not systematically assess whether this was the case before and after the intervention in the current study, so we cannot confirm, using objective measures, whether this occurred in any or all of the pilot hospitals, or conjecture as to whether it contributed to the observed reductions in ET rates. Clinicians do however confirm these practices occurred.

#### Role of agreement to adopt uniform guidelines

It was agreed during the intervention that the AAP (≥35 weeks) and NICE (<35 weeks) thresholds for management would be adopted to promote uniformity of practice, in line with the practice at the Central Women’s Hospital, Yangon. During the initial hospital visits in Myanmar, BOL staff and volunteers noted that hospitals were using different thresholds for phototherapy and ET, and were often performing ET below the recommended thresholds. In discussion with clinicians it became apparent that the lack of efficacious phototherapy encourages clinicians to make conservative decisions and perform ET. For example, if there is little reason to trust the efficacy of the available phototherapy machines, it can make sense to do an ET as a threshold is approached, while the blood bank services are available, rather than waiting a few hours until the threshold is inevitably reached and recalling staff to the hospital premises.

When considered in this context, the provision of effective phototherapy facilitates compliance with guidelines. It is plausible that compliance will increase over time as clinicians adjust to a new clinical reality, where rapidly increasing TSB can be treated without ET.

### Limitations

There are a number of limitations in the current study. Some limitations relate to the fact that this study was implemented as unfunded operational research, reliant on data collected by hospital staff while performing their clinical and administrative roles. BOL did not employ in-country staff with a primary role of supporting hospitals with data-collection until late 2012. A number of other specific limitations are discussed below.

First, pre-intervention evaluation data was collected according to local convention rather than external definition, so it was considered inappropriate to impose an external definition that applied only to the post-intervention period. While ET for hyperbilirubinaemia (the numerator) is straightforward, there was important variation in the definition of the number of jaundiced neonates (the denominator). At Inborn Hospital A, the Annual Report reported the total number of neonates receiving phototherapy regardless of the reason for admission; at other hospitals, the number of admissions for jaundice was based on a review of the NCU Admission Register. To our knowledge, the denominator definitions did not change markedly in the pre- and post-intervention periods so, while the ET rates of the four hospitals may not be directly comparable, the presented relative risk estimates are believed to be unbiased.

Second, when the NCU Annual Report at Outborn Hospital A was found to be inaccurate in the recorded number of ETs for 2011, a retrospective review of the Admission and ET Registers was undertaken. The relative risk was calculated by comparing the ET rate for the 6 months July–December 2011 as the pre-intervention period and the same 6 months of 2012 as the post-intervention period. Full-year data for 2011 could not be collected as the available ET Register was initiated in June 2011. In this hospital, as in the other three pilot hospitals, the intervention took place in late November 2011, but data for late November and all December 2011 were included as part of the pre-intervention period. We consider this to be an appropriate designation as we consider November and December 2011 to be a ‘wash-in’ period, during which new treatment protocols were rolled out in the NCUs. Nevertheless, the fact of having LED phototherapy during this 5 week-period has the effect of making the pre- and post-intervention periods more similar, potentially reducing the size of the estimated relative risks reported here.

Third, Outborn Hospital B saw a one third reduction in total admissions and halving of the number of neonates admitted for phototherapy. This occurred because a larger, better-resourced paediatric hospital opened in the same catchment during the post-intervention period. It is plausible that the reduction of admissions reflected the self-referral of sicker neonates to the new hospital. If so, the average acuity of jaundiced patients in the post-intervention period may be lower than in the pre-intervention period, but we lack individual patient data for the two periods which could support or refute this hypothesis. Comparing the two paediatric hospitals included in this study, however, shows that they had similar results (ET rates reduced from roughly 30 % pre- to 20 % post-intervention), and patient profiles.

Fourth, the LED Treatment Register data presented in this report does not represent the entire cohort of neonates receiving phototherapy during the post-intervention period. No data was collected on neonates treated exclusively with conventional phototherapy. Clinicians indicate that the subset of neonates reported here are likely to be the neonates with higher TSB values, or who were otherwise considered to be at higher risk. We do not believe this impacts our interpretations in any significant way.

Fifth, and finally, we note that for simplicity of data collection the LED Treatment Register recorded date at start and end of treatment and calculated duration of treatment in ‘days’ by subtracting start date from end date; clearly, it would have been more accurate to count hours, but this was not done as it would have increased the reporting load on clinicians.

## Conclusions

We report on a simple intervention at four hospitals comprising the provision, to each hospital, of two LED phototherapy machines to improve the irradiance provided for treatment of jaundice, one photo radiometer to allow hospitals to triage neonates to the most effective conventional phototherapy machines and to allow them to ensure high output from conventional machines, and training in the use of standard guidelines for management of hyperbilirubinaemia. This intervention led to a one-third reduction in the ET rate for outborn neonates and a reduction by two-thirds in the ET rate for inborn neonates in one hospital, and no change in the other; a supplementary analysis, with a new post-intervention period defined *post hoc*, showed both inborn NCUs achieved a two-thirds reduction in ET after provision of additional high intensity phototherapy machines.

An important lesson from the current pilot was that provision of two LED machines was sufficient to make a difference in a hospital with fewer than 400 annual admissions for jaundice, but failed to have an impact in a hospital with over 800 annual admissions for jaundice. We hypothesise that this was because neonates were being removed from LED phototherapy before the completion of their treatment due to high demand for machines. In line with the remedial action taken in the current study, the provision of one LED machine per 150–200 annual admissions for jaundice treatment is recommended. If this is not feasible, the current data suggest that it is preferable to ensure complete treatment on a subset of high risk neonates, to prevent ET in that subset, rather than partial treatment on multiple neonates, which risks no benefit in terms of ET averted.

## Abbreviations

AAP: American Academy of Pediatrics
ABE: Acute Bilirubin Encephalopathy
BOL: Breath of Life program
CI: confidence interval
ET: exchange transfusion
NCU: Neonatal Care Unit
NS: not statistically significant
RR: relative risk ratio
RR_MH_: Pooled (Mantel-Haenszel) relative risk ratio
TSB: Total Serum Bilirubin
